# New Trends of Substance Abuse During COVID-19 Pandemic: An International Perspective

**DOI:** 10.3389/fpsyt.2020.00700

**Published:** 2020-07-16

**Authors:** Simona Zaami, Enrico Marinelli, Maria Rosaria Varì

**Affiliations:** ^1^ Department of Anatomical, Histological, Forensic and Orthopedic Sciences, Sapienza University of Rome, Rome, Italy; ^2^ National Centre on Addiction and Doping, Istituto Superiore di Sanità, Rome, Italy

**Keywords:** substance abuse, COVID-19 pandemic, opioids, new benzodiazepines, new psychoactive substances

## Introduction

In the late 2019, an epidemic of cases with severe acute respiratory syndrome coronavirus 2 (SARS-Cov-2) has spread from China to the rest of the world, resulting in a global pandemic (COronaVIrus Disease 19, COVID-19 pandemic). Starting from the first months of 2020, several restrictions have been imposed by governments to face the public health threat, impacting the usual patterns of drug abuse throughout the world (1). The temporary border closure affected the usual illicit drug route of shipping from country to country, resulting in scarcity of classic street drugs (2). Moreover, restrictive measures internationally adopted by several countries made necessary to close all the usual recreational settings in which stimulants drugs are commonly abused. On the contrary, since in house drugs abuse became the most feasible option, other private encounters might have caught on, such as chemsex (3). In particular this phenomenon, which originated mainly in the large cities of Northern Europe, has gradually spread across the continent and is now a worrying reality in western European countries. Other rising trends of substance abuse include cognitive enhancers and new psychoactive substances (4, 5). Furthermore, the consequent social isolation and the likely limited access to detoxification centers caused additional psychological distress, pushing drug addicts toward alternative psychotropic drugs, possibly through illegal online marketplaces. An international overview of the new trends of drug abuse during the current COVID-19 pandemic and the related health risks are hereby discussed, taking into consideration different points of view.

### Can New Trends of Substance Abuse Be Identified During COVID-19 Pandemic?

As we write this opinion paper, the social and economic restrictions due to the coronavirus pandemic have already seriously impacted health and social fields. COVID-19 outbreak has led to the implementation of social distancing to contain the spread of the disease, changing people’s lifestyle. People have been going through a moment of anxiety and fear for their health and their jobs, and they are forced to live an unfamiliar lifestyle, deprived of relationships. Furthermore, the condition of people with psychological troubles may have worsened during the pandemic as a result of the unconsciously mirroring of others feelings (6). This peculiar situation may have pushed more people toward a deviant behavior linked to licit or illicit substance use, and it may have been a good opportunity for drug dealers to attract new customers. However, global issues have not favored the usual trade business. Indeed, social distancing has substantially reduced drug trafficking on the streets, pushing consumers toward illegal markets on the dark web or through messaging applications (7). Furthermore, the paucity of classic drugs, together with the impossibility to go out to look for those, might have induced addicts to misuse psychoactive prescription drugs such as benzodiazepines (8–10). In this concern, although there is limited scientific evidence, the impact of the COVID-19 pandemic could lead to substantial modifications in substance use patterns, and an increased risk of substitution, adulteration, contamination, and dilution with a potentially harmful substance. As such, reports from forensic science and toxicology laboratories are crucial for the early detection and response to such events (11, 12). Moreover, in this period of home confinement, users might no longer be looking for “socializing” substances to be used in recreational settings, but for psychotropic drugs to be consumed in solitude.

Even short periods of isolation and loneliness can have negative consequences on physical and mental well-being. The feeling of isolation can lead to anxiety and anger, and even sleep disorders, depression, and post-traumatic stress disorders, which may be underestimated due to the lack of specific screening tools (13, 14). Moreover, psychiatric assistance from health professionals is not assured due to the temporary monopolization of psychiatric facilities for COVID-19 treatment (15). In addition to drug addicts using prescription sedatives available at home, some may have shifted to narcotics such as new synthetic opioids or designer benzodiazepines, available online. Indeed, these two classes of new psychoactive substances showed the highest consumption increase in 2019 (16–19).

### COVID-19 Health Risks Associated to Psychotropic Drug Use

The European Monitoring Centre for Drugs and Drug Addiction (18), in Europe, and the National Institute on Drug Abuse (20), in US, first sounded the alarm, raising concerns about the vulnerability of people with substance use disorders to COVID-19, especially because of opiates (e.g. heroin), synthetic opioids, and methamphetamine effects on the respiratory system and pulmonary health (21–23). Comorbidities, including cardiovascular and other respiratory diseases, have proven to worsen prognosis in patients with other coronaviruses affecting the respiratory system, such as SARS-CoV and MERS-CoV (24).

COVID-19 affects the respiratory tract and has a high mortality rate among elderlies and people with comorbidities such as diabetes, cancer, and breathing difficulties. Given the high prevalence of chronic diseases among drug users, many may have been at risk of respiratory distress and death if infected with COVID-19 (20). It is also worth mentioning that smoking heroin or crack cocaine addicts may undergo asthma and chronic obstructive pulmonary disease (COPD) (24). Moreover, people using high doses of prescription opioids or presenting opioid use disorder experience additional challenges for their respiratory health. Indeed, opioids act on the central nervous system with respiratory-depressant effects, and high doses may cause severe hypoxemia, which may lead to irreversible brain damage. Chronic respiratory diseases are already known to increase overdose mortality in opioid users, and reduced lung function due to COVID-19 could similarly threaten this population. There is also a high incidence of cardiovascular diseases among opiates, opioids, and cocaine users (25, 26).

## Discussion

At this time of crisis, the rapid implementation of extraordinary changes is not something “obvious” and “automatic”, but requires a strong effort of adaptation and the active participation of all people, including drug users. Some may be better at withstanding a quarantine for many reasons, including people’s personality. However, being in quarantine can be challenging for addicts, especially substance addicts. Forced isolation and difficulties to move around and obtain illegal substances can impact the behavior of drug abusers. As an example, reports of people violating the quarantine in search of drugs have multiplied in several Italian cities (27). Moreover, the psychological impact of quarantine may have exacerbated a number of mental health problems. Addictions are already a manifestation of psychological discomfort and these circumstances may have worsened psychophysical well-being. In terms of public mental health, the main psychological risk is high stress and anxiety (28). However, due to new and increasingly stringent measures and their effects on many people’s lifestyle and wellness, an increase in alcohol and drug abuse is expected. Depression and self-harm behaviors leading to suicide have been also anticipated. Additionally, new obstacles for obtaining drugs will emerge, worsening the troubles of drug addicts. The current crisis prevents illicit drug trafficking on the streets and imposes the use of alternative methods for obtaining drugs *via* the Internet through specialized websites, and their subsequent shipment by private couriers. Hence, an increase in cannabis product online sales was recorded during the first 3 months of 2020 (29). In the authors’ opinion, a straightening of postal police controls should prevent the spread of this phenomenon. As already mentioned, since recreational drug use usually occurs in groups or crowded environments, the implementation of social distancing in response to the COVID-19 crisis may have modified drug use patterns: a shift to substances that can be consumed in solitude and have a relaxing effect, such as opioids, new synthetic opioids, or new benzodiazepines, is expected (18, 20, 25). In addition, a potentially reduced access to legal substitution treatments is of concern to drug addicts and drug addicts services pushed for an easier access to drugs such as methadone and buprenorphine to help alleviate withdrawal symptoms, reduce drug craving, and prevent opioid overdose (30). In fact, social distancing could also increase the likelihood of isolated overdose and subsequent failure to administer naloxone by health services, potentially causing more deaths. During the pandemic, it may be necessary to suspend or reduce the number of face-to-face meetings and implement alternatives. In our opinion, the continuous operation of drug treatment services, including the continuous supply of substitute therapies and other essential drugs and the implementation of contingency plans to address any shortage of therapies and tools, should be ensured. Before the pandemic, patients receiving methadone had to follow an approved treatment program for opioid addicts, under which the drug could only be administered daily and under supervision. This may not be possible at this time. Patients under opioid addiction treatment with a reasonable degree of stabilization should obtain several doses of methadone in sufficient quantity for several days or refill their buprenorphine prescription over the phone (26). In the opinion of the authors, the public health community should also focus efforts on the development of virtual support meetings for people with psychiatric disorders or undergoing addiction therapy and the possibility to take home medication (31, 32). In addition, it is worth noting that there is a high prevalence of HIV infections, viral hepatitis infections, and liver cancer among intravenous drug users, leading to a weakened immune system. Therefore, the current health crisis could limit access to healthcare, putting this population at risk for many diseases, as hospitals and clinics are already stretched to their maximum capacity (15). These people, who are already stigmatized and underserved by the health system, could therefore face even greater barriers to treatment, increasing their chances of falling ill and being rejected by charities, forcing them to live on the streets or in squats. Self-isolation, required by lockdown and subsequent movements limitation, for homeless drug addicts can be problematic, as they have no choice but to spend time in public spaces with limited personal hygiene, increasing the risk of infection with COVID-19. Addressing the needs of homeless or unstable drug users is important. The efforts of not-for-profit organizations and associations could help in the short term, but they also must address the increasingly stringent measures dictated by governments and closely monitor the safety of their workers.

To conclude, as suggested by the US National Institute on Drug Abuse and the European Monitoring Centre for Drugs and Drug Addiction, a range of resources has to be developed to support situational awareness and inform relevant and timely actions for preparedness and response activities at national and international level related to the impact of the pandemic on the drug situation and eventual new trends of drug abuse. Psychiatric and psychological assistance to addicts undergoing substitution therapy should be implemented through any possible alternative mean during COVID-19 pandemic.

## Author Contributions

SZ provided initial idea and construct of the opinion. EM and MV co-authored and edited the manuscript.

## Conflict of Interest

The authors declare that the research was conducted in the absence of any commercial or financial relationships that could be construed as a potential conflict of interest.
